# Role of collecting duct principal cell NOS1β in sodium and potassium homeostasis

**DOI:** 10.14814/phy2.15080

**Published:** 2021-10-19

**Authors:** Kelly A. Hyndman, Elena Isaeva, Oleg Palygin, Luciano D. Mendoza, Aylin R. Rodan, Alexander Staruschenko, Jennifer S. Pollock

**Affiliations:** ^1^ Department of Medicine Division of Nephrology Section of Cardio‐Renal Physiology and Medicine University of Alabama at Birmingham Birmingham Alabama USA; ^2^ Department of Cellular Biology, Neurobiology and Anatomy Medical College of Wisconsin Milwaukee Wisconsin USA; ^3^ Division of Nephrology Department of Medicine Medical University of South Carolina Charleston South Carolina USA; ^4^ Molecular Medicine Program University of Utah Salt Lake City Utah USA; ^5^ The Department of Internal Medicine Division of Nephrology and Hypertension University of Utah Salt Lake City Utah USA; ^6^ The Department of Human Genetics University of Utah Salt Lake City Utah USA; ^7^ The Medical Service Veterans Affairs Salt Lake City Health Care System Salt Lake City Utah USA; ^8^ Department of Molecular Pharmacology and Physiology University of South Florida Tampa Florida USA; ^9^ The James A. Haley Veterans Hospital Tampa Florida USA

**Keywords:** collecting duct, high salt, Kcnj10, Kcnj16, kidney, K_ir_ channels, nitric oxide, NOS1β, potassium

## Abstract

The nitric oxide (NO)‐generating enzyme, NO synthase‐1β (NOS1β), is essential for sodium (Na^+^) homeostasis and blood pressure control. We previously showed that collecting duct principal cell NOS1β is critical for inhibition of the epithelial sodium channel (ENaC) during high Na^+^ intake. Previous studies on freshly isolated cortical collecting ducts (CCD) demonstrated that exogenous NO promotes basolateral potassium (K^+^) conductance through basolateral channels, presumably K_ir_4.1 (*Kcnj10)* and K_ir_5.1 (*Kcnj16*). We, therefore, investigated the effects of NOS1β knockout on K_ir_4.1/K_ir_5.1 channel activity. Indeed, in CHO cells overexpressing NOS1β and K_ir_4.1/K_ir_5.1, the inhibition of NO signaling decreased channel activity. Male littermate control and principal cell NOS1β knockout mice (CDNOS1KO) on a 7‐day, 4% NaCl diet (HSD) were used to detect changes in basolateral K^+^ conductance. We previously demonstrated that CDNOS1KO mice have high circulating aldosterone despite a high‐salt diet and appropriately suppressed renin. We observed greater K_ir_4.1 cortical abundance and significantly greater K_ir_4.1/K_ir_5.1 single‐channel activity in the principal cells from CDNOS1KO mice. Moreover, blocking aldosterone action with in vivo spironolactone treatment resulted in lower K_ir_4.1 abundance and greater plasma K^+^ in the CDNOS1KO mice compared to controls. Lowering K^+^ content in the HSD prevented the high aldosterone and greater plasma Na^+^ of CDNOS1KO mice and normalized K_ir_4.1 abundance. We conclude that during chronic HSD, lack of NOS1β leads to increased plasma K^+^, enhanced circulating aldosterone, and activation of ENaC and K_ir_4.1/K_ir_5.1 channels. Thus, principal cell NOS1β is required for the regulation of both Na^+^ and K^+^ by the kidney.

## Graphical abstract

Principal cell NOS1β is critical for regulation of both sodium and potassium transport.

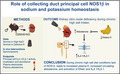

## 1 INTRODUCTION

In society, the likelihood of high sodium (Na^+^) intake throughout life is rising. Although it is debated what an appropriate amount of dietary Na^+^ should be for an individual (O'Donnell et al., 2015), it is clear that many individuals suffer adverse events from chronic dietary salt excess (Polonia et al., 2016; Wong et al., 2017). We reported that kidney principal cell nitric oxide synthase‐1 (NOS1) is critical for maintaining fluid–electrolyte balance and blood pressure control during high‐salt feeding (Hyndman et al., 2013). NOS1 is expressed as alternative splice variants in the kidney; NOS1α full length, NOS1β, and NOS1γ N‐truncated proteins. In renal medullary homogenates from mice and human subjects, NOS1β is predominantly expressed compared to NOS1α (Hyndman et al., 2013, 2016). Moreover, SNPs in the human NOS1 gene correlate with hypertension (Iwai et al., 2004; Padmanabhan et al., 2010) and low urinary NO metabolites (NOx) excretion may be predictive of hypertension and/or kidney disease (Armas‐Padilla et al., 2007; Baumann et al., 2011; Hsu & Tain, 2019; Imanishi et al., 2013; Kemmner et al., 2017). Mice with a genetic deletion of NOS1 splice variants from the principal cell in the kidney excrete significantly less urinary NOx and display a rightward‐shift in the pressure‐natriuresis relationship (Hyndman et al., 2013). However, on a high‐salt diet, NO‐deficient mice fail to appropriately suppress the Na^+^‐retaining hormone, aldosterone, despite suppressed renin activity. As a consequence, NO deficient mice have greater activity of the epithelial sodium channel (ENaC) (Hyndman et al., 2015, 2017). The mechanisms underlying the elevated aldosterone levels in NO deficient mice are incompletely understood.

The plasma potassium (K^+^) level is normally maintained between 3.5 and 5.0 mEq/L. K^+^ homeostasis is precisely regulated by the kidney, particularly by the renal cells in the distal nephron that regulate urinary K^+^ excretion (Ellison et al., 2016). In the cortical collecting duct (CCD) principal cell, K^+^ is transported by the Na^+^/K^+^‐ATPase, resulting in high intracellular K^+^ concentrations that contribute to the electrochemical gradient driving K^+^ secretion (Welling, 2016). On the apical membrane, sodium reabsorption through ENaC generates a lumen‐negative transepithelial potential that also drives K^+^ secretion through the ROMK (K_ir_1.1) and the big conductance K^+^ (BK) channels (Bailey et al., 2006; Wang & Giebisch, 2009; Welling, 2016). On the basolateral membrane, K^+^ recycling occurs through the basolateral homo‐ and heteromeric K_ir_4.1 and K_ir_4.1/K_ir_5.1 channels (Manis et al., 2020; Su et al., 2019). Recent preclinical studies have shown that the basolateral K_ir_4.1/K_ir_5.1 channel is critical for renal salt handling and blood pressure control (Cuevas et al., 2017; Palygin, Levchenko, et al., 2017; Palygin et al., 2017; Penton et al., 2020; Tomilin et al., 2018; Wang, Su, et al., 2018). The importance of basolateral K^+^ channels is also reinforced by clinical data that identify loss of function mutations in *KCNJ10* (K_ir_4.1) causing EAST/SeSAME syndrome, in which patients have hypokalemia, hypomagnesemia, and salt‐wasting (Celmina et al., 2019). Similarly, recent studies revealed that mutations in *KCNJ16* (K_ir_5.1) cause a novel tubulopathy with hypokalemia, salt‐wasting, and disturbed acid‐base homeostasis (Schlingmann et al., 2021). Previous electrophysiological studies determined that CCD basolateral K^+^ channel activity was decreased with inhibition of NOS and this was reversed with exogenous NO (Lu & Wang, 1996). These data suggest that NOS‐mediated NO stimulates basolateral K^+^ channel activity. Thus, the purpose of the current study was to test the hypothesis that principal cell NOS1β modulates K_ir_4.1/K_ir_5.1 activity under high Na^+^ dietary conditions.

## 2 METHODS

### 2.1 Chinese Hamster Ovary K_ir_4.1/K_ir_5.1 single‐channel recordings

To determine if NOS1β modulates K_ir_4.1/K_ir_5.1 activity an in vitro, overexpression approach was used with Chinese hamster ovary (CHO) cells (American Type Culture Collection). CHO cells were maintained under standard culture conditions (DMEM, 10% fetal bovine serum, 50 U/ml penicillin‐streptomycin, 37°C, 5% CO_2_), and transfected with PCDNA3.1+ vectors containing K_ir_4.1, K_ir_5.1, NOS1β, or eGFP using the Polyfect reagent (Qiagen) according to the manufacturer's protocol. Single‐channel recordings of K_ir_4.1/K_ir_5.1 activity were carried out 24 h following transfection with the external buffer being physiological saline solution (PSS, composition [in mM]: 150 NaCl, 5 KCl, 1 CaCl_2_, 2 MgCl_2_, 5 glucose, and 10 HEPES [pH 7.35]) using patch pipettes filled with a solution of the following composition (in mM): 150 KCl, 2 MgCl_2_, and 10 HEPES (pH 7.35) and having resistances of 8–10 MΩ. Patch‐clamp recordings of inwardly rectifying K^+^ currents (~40‐pS conductance) were performed in the cell‐attached voltage‐clamp configuration using an Axopatch 200B amplifier (Molecular Devices) and Digidata 1440A analog‐to‐digital converter (Molecular Devices). Currents were low‐pass filtered at 1 kHz with an eight‐pole Bessel filter (Warner Instruments). The pipette voltage was 60 mV. The active channel number (N) and open probability (P_o_) were determined during baseline recordings and after application of the 1 mM N5‐[imino(nitroamino)methyl]‐l‐ornithine, methyl ester, monohydrochloride (l‐NAME, a NOS inhibitor). Data analysis was performed using pCLAMP 10.6 software. Total K_ir_4.1/K_ir_5.1 activity is represented by *NP_o_*.

### 2.2 Animals

All animal use and welfare adhered to the NIH Guide for the Care and Use of Laboratory Animals following a protocol reviewed and approved by the Institutional Laboratory Animal Care and Use Committees of the University of Alabama at Birmingham (UAB) or the Medical College of Wisconsin. Principal cell, CD‐specific NOS1 knockout male mice (*Aqp2*‐CRE with *Nos1^flox^* ^/^ *^flox^*; CDNOS1KO) and littermate controls (*Nos1^flox^* ^/^ *^flox^*) from the colony maintained at UAB were used (Hyndman et al., 2013, 2015, 2017; Mendoza & Hyndman, 2019). Only male mice were used because our previous publications have established that male CDNOS1KO mice have salt‐sensitive increases in blood pressure and inappropriate ENaC activation (Hyndman et al., 2013, 2015, 2017). All mice were weaned on NIH‐31 diet (Envigo; 7017 (irradiated), 0.3% Na^+^, 0.6% K^+^) and were used in the study at 12–20 weeks of age. Homozygous NOS1^flox/flox^ mice were used as controls and CDNOS1KO mice genotypes were confirmed as previously described (Hyndman et al., 2013, 2015, 2017; Mendoza & Hyndman, 2019). Mice were group‐housed with standard bedding and nesting sheets for enrichment in an animal facility with a 12 h light: 12 h dark cycle.

### 2.3 Dietary interventions and metabolic cage studies

From 2013 until 2020, cohorts of littermate mice were randomly assigned to the following interventions:

#### 2.3.1 Intervention 1: Dietary sodium challenges

Mice were housed in standard cages and switched from standard NIH‐31 diet to the following purified diets (Envigo):

1. Normal Na^+^, normal K^+^ (NSNK; TD.88238 (non‐irradiated, powder), 0.74% NaCl (0.3% Na^+^), 1% K^+^)
2. High Na^+^, normal K^+^ (HSNK; TD.88238 supplemented with NaCl to make, 4.0% NaCl (1.6% Na^+^), 1% K^+^)

Mice remained on these diets for 7 days. At dissection, between 9 and 10 a.m., the mice were euthanized, and kidneys excised, de‐capsulated, and half of the left kidney fixed for immunohistological analysis as described below. The right kidney was de‐capsulated, and cortex samples snap‐frozen in liquid N_2_, and stored at −80°C.

#### 2.3.2 Intervention 2: Spironolactone

Mice were anesthetized with inhaled 2% isoflurane and they received a subcutaneous implant of either spironolactone (25 mg, 21‐day slow‐release) or a vehicle pellet (Innovative Research of America). After surgery, mice were maintained in standard cages on a NS gel diet (TestDiets, microstabilized rodent diet LD101 with 0.16% Na^+^/0.2% K^+^) for 3 days, then switched to a HS diet for 7 days (LD101 with 1.6% Na^+^/0.2 K^+^). A subset of these mice were individually acclimated to metabolic cages for 2 days before 24 h urine collections (under water‐saturated mineral oil) occurred on day 7 of the HS diet. Urine was spun at 1000 *g* for 5 min to pellet any debris and the supernatant stored at −80°C until analysis. At this point, the animals were euthanized and the blood and kidneys were collected. Blood electrolytes were immediately analyzed with EC8+ cartridges and the i‐STAT (Abbot Labs). Urine electrolytes were measured by atomic absorption spectrophotometry (Thermo Fisher). Excretion was calculated by multiplying the concentrations by urine flow.

#### 2.3.3 Intervention 3: Dietary K^+^ restriction

Mice were housed in standard cages and switched from standard diet NIH‐31 diet to the following purified diets (Envigo):

1. Normal sodium, normal potassium (NSNK; TD.88238 (non‐irradiated, powder), 0.3% Na^+^, 0.74% NaCl, 1% K^+^)
2. Normal sodium, deficient potassium (NSDK; TD.88239 (non‐irradiated, powder), 0.3% Na^+^, 0.74% NaCl, 15–30 ppm K^+^)
3. High sodium, normal potassium (HSNK; TD.88238 supplemented with NaCl to make, 4.0% NaCl, 1.6% Na^+^, 1% K^+^)
4. High sodium, deficient potassium (HSDK; TD.88239 supplemented with NaCl to make 4.0% NaCl, 1.6% Na^+^, 15–30 ppm K^+^).

Mice remained on these diets for only 4 days to ensure that plasma K^+^ did not reach critically low concentrations. At this time between 9 and 10 a.m., the mice were anesthetized with 2% isoflurane and blood taken via cardiac puncture with a sodium heparinized syringe (rinsed once and expelled to leave only trace amounts in the syringe). Plasma was isolated (1000 *g*, 10 min, 4°C) and ion concentrations determined with ion selective electrodes (Medica Easylyte). The remaining plasma was snap‐frozen. Kidneys were excised, de‐capsulated, and cortex samples were snap‐frozen. Adrenal glands were excised, cleaned of the surround fat, and snap‐frozen. All tissues and plasma were stored at −80°C. Plasma aldosterone was measured by radio‐immunoassay (Siemens, Coat‐A‐Count Aldosterone).

### 2.4 Isolation renal tubules and single‐channel recordings

Mice were deeply anesthetized with isoflurane and both kidneys were harvested without flushing. Animals were euthanized by pneumothorax. Kidneys were placed into an ice‐cold solution of the following composition (in mM): 119 NaCl, 4.5 KCl, 2 CaCl_2_, 1.3 MgCl_2_, 26 NaHCO_3_, 1.2 NaH_2_PO_4_, 5 glucose, pH 7.35, cleaned from the kidney capsule, and 0.5–1.0 mm slices were cut using a microtome (EMS OTS‐4000). Kidney slices were then transferred to an incubation chamber where they rested in the oxygenated solution until use. For tubule isolation, individual slices were pre‐incubated for 20 min at 37°C in PSS containing 0.7 mg/ml collagenase type I (Alfa Aesar) and 4 mg/ml of dispase II (Roche Diagnostics). Individual CCD tubules were isolated by applying local mechanical vibration (GFG‐8250A, function generator frequency 40–50 Hz; amplitude 20–30 μm) with a flame‐sealed ball‐shaped micropipette (size 0.5 mm) directly to the kidney slice. (Isaeva et al., 2019) Isolated CCDs were placed on cover glass chips (5 × 5 mm) coated with poly‐l‐lysine and transferred to the recording chamber mounted on an inverted Nikon TE 2000‐S microscope. Patch‐clamp recordings and data analyses were performed as outlined above for the CHO cells.

### 2.5 Western blotting and immunohistochemical localization

Western blots were performed with kidney cortical homogenates as previously described in detail (Hyndman et al., 2017; Mendoza & Hyndman, 2019). The primary antibodies for K_ir_4.1 (ab105102, lot GR1925047, a goat polyclonal, Abcam) and K_ir_5.1 (ab74130, lot GR259059‐2, a rabbit polyclonal, Abcam) were used at a 1/1000 dilution and were previously validated against rat knockout tissues (Palygin, Levchenko, et al., 2017; Palygin, Pochynyuk, et al., 2017). For immunohistochemistry, in a subset of control and CDNOS1KO mice on a high‐salt diet, the left kidney was excised, cut in cross‐section, and fixed for 24 h in 10% neutral buffered formalin. Kidneys were then rinsed in 10 mM PBS, dehydrated in an increasing concentration of ethanol, and embedded in paraffin. Kidney sections were cut at 4 μm, dried, and deparaffinized. For antigen retrieval, slides were treated with a citrate buffer (10 mM sodium citrate, 0.05% Tween20, pH 6.0) in a steamer for a total of 35 min. K_ir_4.1 (Alomone Labs, #APC‐035, lot APC035AN1502, knockout tissue validated), K_ir_5.1 (Sigma, #SAB4501636, lot 211083) and ROMK (kind gift of Dr. Paul Welling; validated against knockout tissues [Wade et al., 2011]) were localized using fluorescently tagged secondary antibodies, and co‐expression of the principal cell water channel aquaporin‐2 (AQP2, monoclonal AQP2‐FITC or AQP2‐Alexa Fluor®647, clone E‐2, Santa Cruz Biotechnology) or the DCT marker NCC (polyclonal, NCC‐FITC, #401D‐FITC, Stressmarq). Primary antibodies for K_ir_5.1 and NCC‐FITC were diluted 1/100 in 2.5% normal horse serum (Vector Labs), AQP2‐FITC 1/300, and K_ir_4.1, ROMK, and AQP2‐647 1/1000. Primary antibodies were placed on the hydrated tissue sections for 1 h at room temperature. Secondary antibodies (Alexa Fluor™, Fisher Scientific) were diluted to 1 μg/ml with 2.5% normal horse serum and were placed on the sections for 1 h at room temperature. After two washes in 10 mM PBS, autofluorescence was quenched with the Vector® TrueVIEW® autofluorescence quenching kit for 5 min, then nuclei were stained for 5 min at room temperature in 1 μg/ml Hoechst 33342. Slides were mounted in VECTASHIELD® Vibrance with DAPI Antifade Mounting Medium (Vector Labs). An investigator blinded to the sample key imaged the slides with a Bx43 microscope with X‐Cite® 120LED set to 20% (Lumen Dynamics), fitted with a DP80 camera, and images taken with CellSens Dimension software (v1.12, Olympus). Exposure time, gain (1×), brightness, and contrast were applied uniformly to each sample. These images were then analyzed by an additional investigator blinded to the key, and the mean fluorescent intensity in the CCD principal cell (positive for AQP2) or DCT (positive for NCC) was determined with ImageJ (v2.1.0/1.53c). The red, green, and blue channels of the image were split, and the channel of interest was analyzed by setting the threshold to 40 and 255 pixel intensity, outlining the CCD, and analyzing particle mean intensity. Once all values were collected, the groups were unblinded to calculate mean intensity per CCD, and the values reported are normalized to the control mouse group.

### 2.6 PCR for recombined Nos1 alleles

DNA was extracted from inner medullae and adrenal glands using the GenElute™ Mammalian Genomic DNA Miniprep Kit following the manufacturer's instructions (Sigma). Standard PCR was run using DreamTaq Hot Start Green PCR Master Mix 2x (Thermo Scientific) as previously described in detail (Mendoza & Hyndman, 2019).

### 2.7 Statistics

Data are reported as mean ± standard error of the mean in the tables. In figures, all values are presented in a box plot with the box reporting the interquartile range, the horizontal line at the median, and the whiskers representing the minimum and maximum values. To test for a significant difference among means, a two‐factor ANOVA (genotype, spironolactone drug/diet) was performed and Tukey's multiple comparison post hoc test was used. For metabolic cage data, a repeated measures two‐factor ANOVA (genotype, diet) and Tukey's multiple comparison post hoc test was used. For dichotomous comparisons, Student's unpaired or paired two‐tailed *t*‐tests or Mann–Whitney nonparametric tests were performed (α = 0.05). Statistics were done using Prism 9.0.0 (GraphPad).

## 3 RESULTS

### 3.1 NOS1β regulation of basolateral Kir4.1/Kir5.1 channels

**FIGURE 1:**
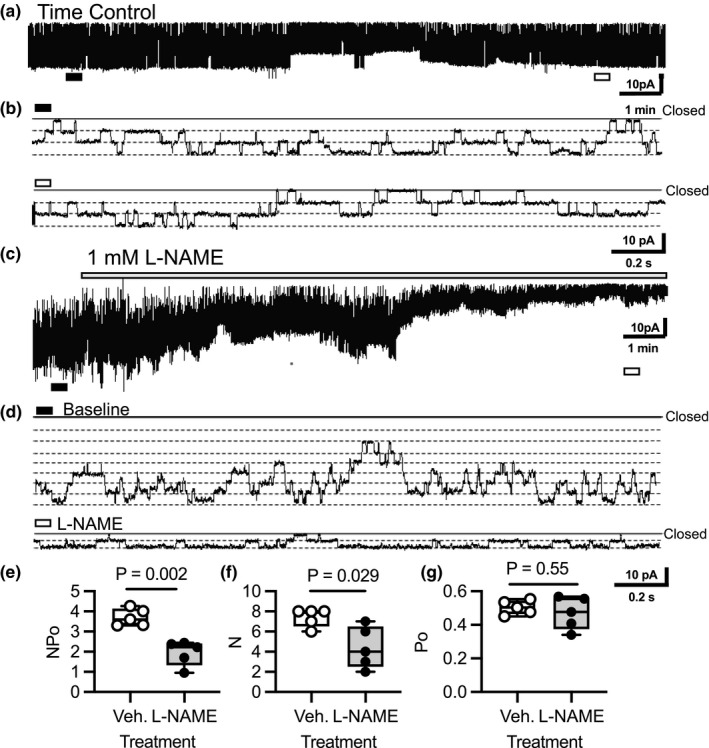
Effects of NOS1β inhibition with L‐NAME on K_ir_4.1/K_ir_5.1 single‐channel activity in CHO cells overexpressing K_ir_4.1, K_ir_5.1, and NOS1β. (a) A representative time control current trace of 40‐pS K^+^ channel (K_ir_4.1/K_ir_5.1) single‐channel activity over the course of the experiment. Patches were clamped to –Vp = −60 mV. (b) Examples of single‐channel activity on an expanded time scale. The black and white bars represent the expanded time scale. Each dashed line in the magnified images represents a single‐channel opening state (number of active channels). (c) A representative current trace of K_ir_4.1/K_ir_5.1 single‐channel activity taken before and following administration of 1 mM L‐NAME. (d) Examples of single‐channel activity on an expanded time scale. The black bar in (b) represents the expanded time scale during baseline. The white bar represents the expanded time scale at the end of the L‐NAME treatment. (e) Summary for K_ir_4.1/K_ir_5.1 activity (*NP_o_*), (f) number of channels (*N*), and (g) open probability (*P_o_*) are reported (*N* = 5 independent experiments). *p* values from a Student's paired, two‐tailed, *t*‐test are listed in the figure

Nitric oxide modulation of basolateral K^+^ conductance was previously reported in studies using freshly isolated CCDs (Lu et al., 1997; Lu & Wang, 1996). The CD principal cell predominantly expresses the NOS1 splice variant, NOS1β (Hyndman, Arguello, et al., 2016; Hyndman et al., 2013). To determine if NOS1β activity modulates K_ir_4.1/K_ir_5.1 activity, we first used a reconstituted system with single‐channel K_ir_4.1/K_ir_5.1 recordings in CHO cells overexpressing K_ir_ and NOS1β (Figure 1). A representative time control experiment demonstrates K_ir_4.1/K_ir_5.1 activity (*NP_o_*) is maintained over the experimental period (Figure 1a,b). The inhibition of NOS1β with L‐NAME significantly reduced K_ir_4.1/K_ir_5.1 activity by decreasing the number (*N*) of active channels (Figure 1c–g). This is consistent with a stimulatory effect of NO generated by NOS1β on K_ir_4.1/K_ir_5.1 activity as also suggested previously (Lu et al., 1997; Lu & Wang, 1996).

### 3.2 In vivo principal cell K_ir_4.1/K_ir_5.1 activity and localization in the kidney

**FIGURE 2:**
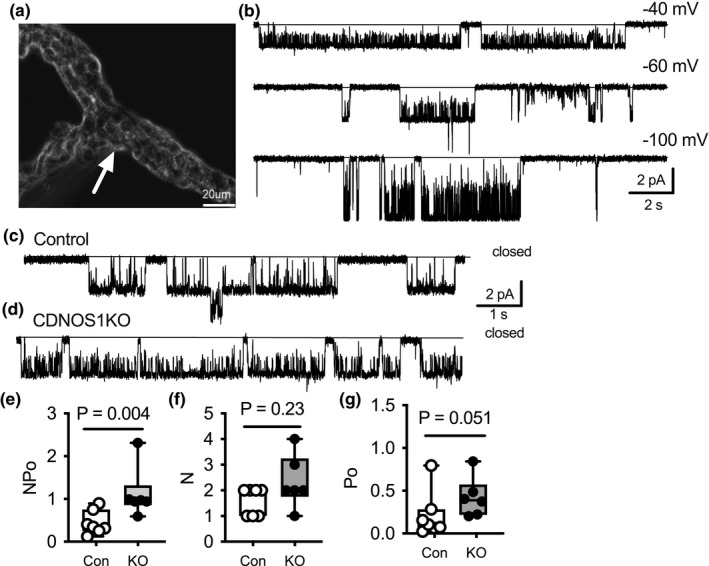
K_ir_4.1/K_ir_5.1 single‐channel activity in the cortical collecting duct (CCD) of control (con) and CDNOS1KO (KO) mice on a 7‐day high‐salt diet (4% NaCl). (a) Representative image of a CCD isolated by the vibrodissociation technique. Arrow points to basolateral patch. Scale bar is 20 µm. (b) Representative current traces of K_ir_4.1/K_ir_5.1 channel recorded from the basolateral membrane of CCD principal cells at different membrane potentials. Solid lines represent the closed state. Examples of single‐channel potassium currents recorded from CCD cells of (c) control and (d) CDNOS1KO mice on high‐sodium/normal potassium diet. Patches were clamped to –Vp = −60 mV. Summary graphs of (e) Kir4.1/Kir5.1 activity (*NP_o_*), (f) active channels (N), and (g) open probability (P_o_). *p* values reported from the Mann–Whitney nonparametric test. *N* = 6–7 independent experiments

A high‐salt diet activates principal cell NOS1β (Hyndman et al., 2013; Hyndman, Dugas, et al., 2016); thus, we predicted that high salt‐fed CDNOS1KO mice would have lower basolateral K^+^ conductance than control mice. Control and CDNOS1KO mice were maintained on a HS diet for 7 days and CCD principal cell single‐channel recordings of the K_ir_4.1/K_ir_5.1 were performed (Figure 2a–d). Surprisingly, CDNOS1KO mice had significantly greater K_ir_4.1/K_ir_5.1 activity compared with control mice (Figure 2b–g). As compared to NO effects on K_ir_4.1/K_ir_5.1 activity in cultured cells, where L‐NAME decreased *N* without an effect on open probability (*Po*), the increased activity of K_ir_4.1/K_ir_5.1 in CDNOS1KO mice was due to increased *Po*. This suggests that in the absence of NOS1β, K_ir_4.1/ K_ir_5.1 is activated by other mechanisms, as discussed further below.

**FIGURE 3:**
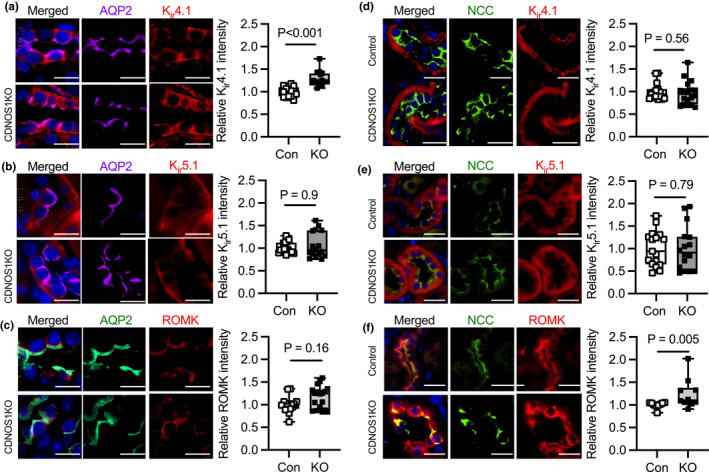
Representative immunofluorescent images of CCD principal cells (anti‐aquaporin‐2, positive, AQP2) and distal convoluted tubules (anti‐sodium chloride cotransporter positive, NCC) expressing K_ir_4.1, K_ir_5.1, or ROMK. Control (Con) and CDNOS1KO (KO) mice were on a 7‐day high‐NaCl diet. Mean intensity normalized to control is reported. Nuclei were stained blue with Hoechst 33342 in the merged image. (a) K_ir_4.1 and (b) K_ir_5.1 were localized to the basolateral membrane of the CCD. (c) ROMK was localized to the apical membrane of the CCD. Co‐localization of NCC and the K^+^ channels: (d) K_ir_4.1, (e) K_ir_5.1, and (f) ROMK. Mean intensity of individual CCDs or DCTs (2–3 each) from *N* = 6 control and CDNOS1KO mice were plotted. Mann–Whitney test *p* values provided. Scale bar = 10 µm

**FIGURE 4:**
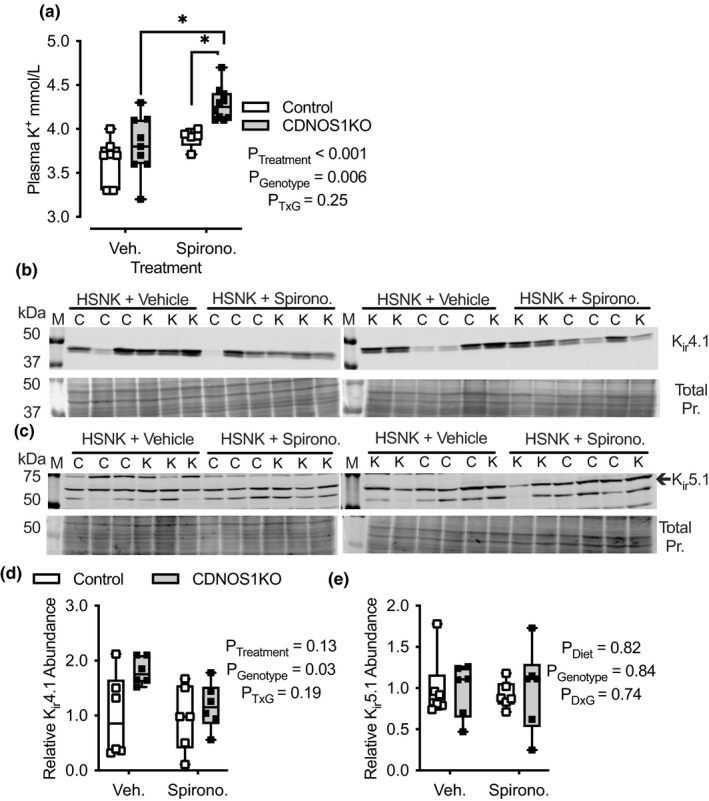
Plasma K^+^ and western blot analysis of cortical lysates from male control and CDNOS1KO mice on day 7 of a high Na^+^, normal K^+^ (HSNK; 1.6% Na^+^/0.3% K^+^) diet with either vehicle (Veh.) or spironolactone (Spirono.). (a) Plasma K^+^, *N* = 6 per genotype. Two‐ Factor ANOVA reported and * represents *p* < 0.05 Tukey's post hoc multiple comparison test. See Table 1 for means ± SEM. (b, c) Renal cortical abundance as determined by western blot of K_ir_4.1 (48 kDa) and K_ir_5.1 (60 kDa) channels from control (C) and CDNOS1KO (K) mice. Total Protein (pr.) stained with Coomassie to demonstrate equal loading of samples. (d) Relative densitometry of the K_ir_4.1 or (e) K_ir_5.1 abundance normalized to the vehicle, control group. *N* = 6 mice in each group. Two‐Factor ANOVA reported and Tukey's post hoc analysis failed to detect any further statistical significance among the groups

We immunolocalized K_ir_4.1, K_ir_5.1, and ROMK in the CCD principal cell from control and CDNOS1KO mice on HS diet. Immunoreactivity to AQP2 was used to identify the CCD principal cell (Figure 3). As expected, K_ir_4.1 and K_ir_5.1 were expressed on the basolateral membrane of the CCD principal cells (Figure 3a,b). K_ir_4.1 mean intensity was significantly greater in the CCD of the CDNOS1KO mice compared to controls (Figure 3a), while K_ir_5.1 mean intensity was not significantly different between the mice (Figure 3b). A change in abundance of K_ir_4.1 but not K_ir_5.1 may affect both heteromeric channels and/or homomeric channel activity. ROMK was expressed on the apical membrane of the CCD principal cell and the mean intensity was not statistically different between the genotypes (Figure 3c). Western blots with cortical homogenates from mice on HS diets confirmed that CDNOS1KO mice had a significantly greater abundance of K_ir_4.1 than control mice but similar levels of K_ir_5.1 (Figure 4).

K_ir_4.1 and K_ir_5.1 are also expressed by the DCT (Wang, Cuevas, et al., 2018). Thus, we immunolocalized K_ir_4.1, K_ir_5.1, and ROMK to the DCT (marked with immunoreactivity to the sodium chloride cotransporter, NCC) (Figure 3d–f). There were no statistically significant differences in K_ir_4.1 (Figure 3d) or K_ir_5.1 (Figure 3e) mean intensity between the control and CDNOS1KO mice. However, ROMK mean intensity was greater in the DCT of the CDNOS1KO mice compared to the control mice (Figure 3f).

### 3.3 Mineralocorticoid receptor antagonism

Aldosterone promotes K_ir_4.1/K_ir_5.1 activity (Tomilin et al., 2018). We previously showed, and confirmed here, that CDNOS1KO mice have elevated aldosterone levels when on a HS diet (Hyndman et al., 2017). We therefore reasoned that the greater K_ir_4.1 abundance and K_ir_4.1/K_ir_5.1 activity seen in the CDNOS1KO mice may be aldosterone‐dependent. Vehicle‐ or spironolactone‐treated control and CDNOS1KO mice consumed similar amounts of Na^+^, K^+^, and water on the HS diet (Table 1). All mice had similar diuresis and natriuresis (Table 1). Plasma Na^+^ was significantly greater in the CDNOS1KO mouse on the HS gel diet, confirming earlier reports (Hyndman et al., 2017) (Table 1). This genotypic difference was negated with spironolactone treatment (Table 1). In the two‐way ANOVA, there were statistically significant effects on plasma K^+^ of both spironolactone (*P*_treatment_ < 0.001) and CDNOS1KO (*P*_genotype_ = 0.006) without a significant interaction (*P*_treatment_ _× genotype_ = 0.25). In post hoc testing, plasma K^+^ was greater in spironolactone‐treated CDNOS1KO compared to control (*p* < 0.05, Tukey's multiple comparison test) (Figure 4a, Table 1). These results indicate that aldosterone upregulation is required in the CDNOS1KO mice to maintain potassium homeostasis. K_ir_4.1 abundance was significantly higher in the CDNOS1KO mice compared to control (Figure 4b,d), and although spironolactone‐treated CDNOS1KO mice had lower K_ir_4.1 abundance this did not reach statistical significance (Figure 4b,d). There were no statistically significant differences in K_ir_5.1 abundance in the mice between vehicle and spironolactone treatment (Figure 4c,e).

**TABLE 1:** Metabolic cage intake, output, and plasma values for control and CDNOS1KO mice treated with vehicle or spironolactone on a 7‐day high‐salt diet

	Placebo		Spironolactone		Two‐Factor ANOVA		
	Control	CDNOS1KO	Control	CDNOS1KO	*P*_Treatment_	*P*_Genotype_	*P*_T × G_
Sample size	6	6	7	7			
Mouse mass (g)	27.2 ± 0.9	29.0 ± 0.9	30.1 ± 2.2	28.8 ± 1.0	0.28	0.82	0.21
Food intake (g/day)	9.7 ± 0.5	9.9 ± 0.6	9.6 ± 1.1	10.9 ± 0.5	0.56	0.27	0.45
K intake (mg/day)	18.8 ± 1.1	19.7 ± 0.9	18.7 ± 2.1	21.2 ± 1.0	0.60	0.21	0.54
Na intake (mg/day)	155.1 ± 8.6	159.1 ± 9.0	153.4 ± 17.1	174.0 ± 7.9	0.56	0.27	0.45
Water intake (ml/day)	11.5 ± 0.7	13.8 ± 0.6	12.9 ± 1.5	11.0 ± 1.4	0.56	0.88	0.07
UV (ml/day)	8.2 ± 0.5	8.0 ± 1.2	9.7 ± 1.0	6.6 ± 0.9	0.92	0.08	0.14
UNaV (mmol/day)	4.8 ± 0.3	4.3 ± 0.3	4.3 ± 0.3	4.3 ± 0.4	0.10	0.77	0.82
UKV (mmol/day)	0.3 ± 0.01	0.3 ± 0.03	0.22 ± 0.03	0.26 ± 0.02	**<0.001**	0.34	0.51
PNa (mmol/l)	142.2 ± 1.0	145.2 ± 1.1*	142.3 ± 0.7	143.8 ± 0.03	0.45	**0.01**	0.35
PK (mmol/l)	3.6 ± 0.1	3.8 ± 0.1	4.0 ± 0.09	4.3 ± 0.07*	**<0.001**	**0.018**	0.43
PCl (mmol/l)	115.0 ± 1.7	114.6 ± 1.1	120.8 ± 0.8*	116.9 ± 0.6	**0.002**	0.073	0.15

Note

Mean ± standard error of the mean reported. Bold represents significant group effects while * represents *p* < 0.05 between control and CDNOS1KO from Tukey's post hoc analysis.

### 3.4 Dietary K^+^ restriction

**FIGURE 5:**
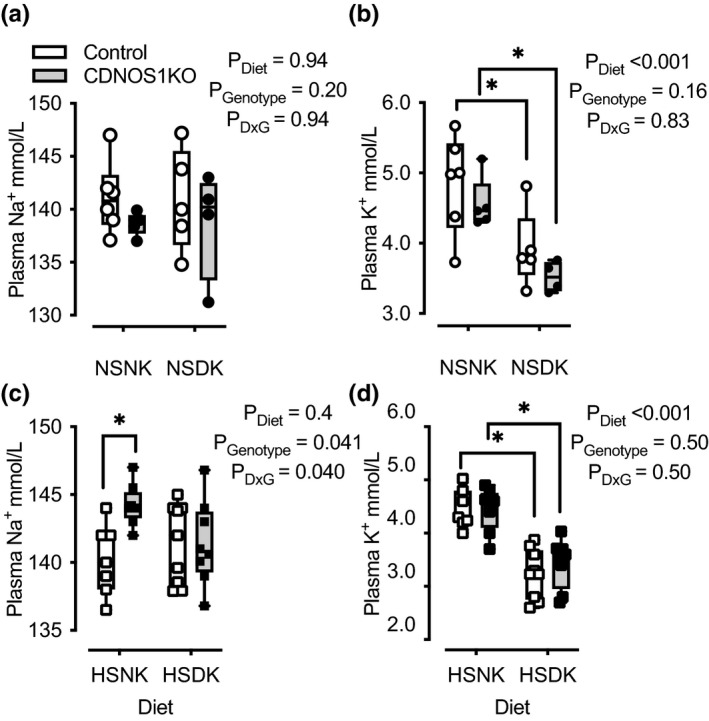
Plasma Na^+^ and K^+^ from male control and CDNOS1KO mice on various Na^+^ and K^+^ purified chow diets for 4 days. (a) Plasma Na^+^ was similar among the groups: normal Na^+^/normal K^+^ (NSNK: 0.3% Na^+^, 1% K^+^), normal Na^+^/deficient K^+^ (NSDK: 0.3% Na^+^, 0.0001% K^+^). *N* = 4–6. (b) Plasma K^+^ was significantly reduced in both genotypes after 4 days of NSDK diet and did not differ between genotypes. *N* = 4–6. (c) On a high‐Na^+^ diet with normal K^+^ (HSNK; 1.6% Na^+^, 1% K^+^), CDNOS1KO mice had significantly greater plasma Na^+^, which was reversed by K^+^‐deficient diet. *N* = 7–8. (d) Plasma K^+^ was significantly reduced by 4 days of high‐Na^+^ /deficient K^+^ diet (HSDK; 1.6% Na^+^, 0.0001% K^+^), without differences between genotypes. *N* = 8–9. Two‐Factor ANOVA reported with * represents *p* < 0.05 from the Tukey's multiple comparison test

We reasoned that if the aldosterone elevation in high salt‐fed CDNOS1 knockout mice (Hyndman et al., 2017) is required to maintain potassium homeostasis, low‐potassium diet should normalize aldosterone levels. Control and CDNOS1KO mice on day 4 of a NSDK (normal Na^+^/deficient K^+^) purified diet had similar plasma Na^+^ (Figure 5a), but as expected significantly reduced plasma K^+^ (Figure 5b). However, 4 days of a HSNK diet resulted in significantly greater plasma Na^+^ in the CDNOS1KO mouse compared to controls (confirming our previous findings [Hyndman et al., 2017]). This genotype difference was abolished with a HSDK diet (Figure 5c). Plasma K^+^ was significantly reduced in both groups of mice on a HSDK diet (Figure 5d).

**FIGURE 6:**
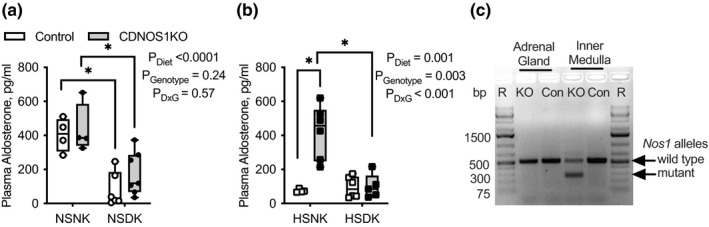
Plasma aldosterone from male control and CDNOS1KO mice on various Na^+^ and K^+^ diets for 4 days. (a) Plasma aldosterone concentration is similar between the genotypes on the normal Na^+^/deficient K^+^ (NSNK: 0.3% Na^+^/1% K^+^) and normal Na^+^/deficient K^+^ (NSDK:0.3% Na^+^,0.0001%K^+^). Deficient K^+^ diet significantly reduced plasma aldosterone in both genotypes. *N* = 4–7 mice per group. (b) Plasma aldosterone from mice on a high Na^+^, normal K^+^ (HSNK, 1.6% Na^+^/1% K^+^), or high Na^+^, deficient K^+^ diet (HSDK, 1.6% Na^+^, 0.0001% K^+^). CDNOS1KO mice have greater plasma aldosterone on a HSNK diet; however, this genotype difference was abolished in the mice on a HSDK diet. HSNK *N* = 4–6 mice per group. Two‐Factor ANOVA reported with * represents *p* < 0.05 from the Tukey's multiple comparison test. (c) Agarose gel image of the PCR products of genomic DNA amplifying wildtype alleles (500 base pairs, bp) or the mutant recombined alleles (250 bp) of *Nos1* from the adrenal gland and inner medulla samples from control (Con) and CDNOS1KO (KO) mice. Although there is genomic recombination in the inner medulla, there was no detectable recombination in the adrenal gland. Thus, *NOS1* DNA is not mutated in the adrenal gland. R‐ molecular ruler

Plasma aldosterone from these mice followed a similar pattern as plasma Na^+^ (Figure 6). All mice on a NSDK diet had significantly lower plasma aldosterone compared to mice on a NSNK diet (Figure 6a). However, CDNOS1KO mice on a HSNK diet had significantly greater plasma aldosterone than control mice (Figure 6b). This HS genotypic difference was eliminated with a HSDK diet (Figure 6b). Thus, low dietary K^+^ normalizes aldosterone and prevents the mild increase in plasma Na^+^ in the CDNOS1KO mouse during high Na^+^ intake.

Recent studies determined that AQP2 may be expressed in tissues other than the principal cell and male reproductive system, leading to the deletion of floxed genes in unintended tissues in AQP2‐Cre positive mice (Mendoza & Hyndman, 2019; Ramkumar et al., 2018). No mutant *Nos1* alleles were detected in the adrenal glands of any mice, yet as a positive control, kidney inner medulla of the CDNOS1KO mouse did have the expected mutated *Nos1* allele (Figure 6c). These data show that the effects of CDNOS1KO on aldosterone production are not from unintentional manipulation of the *Nos1* alleles in the adrenal gland.

**FIGURE 7:**
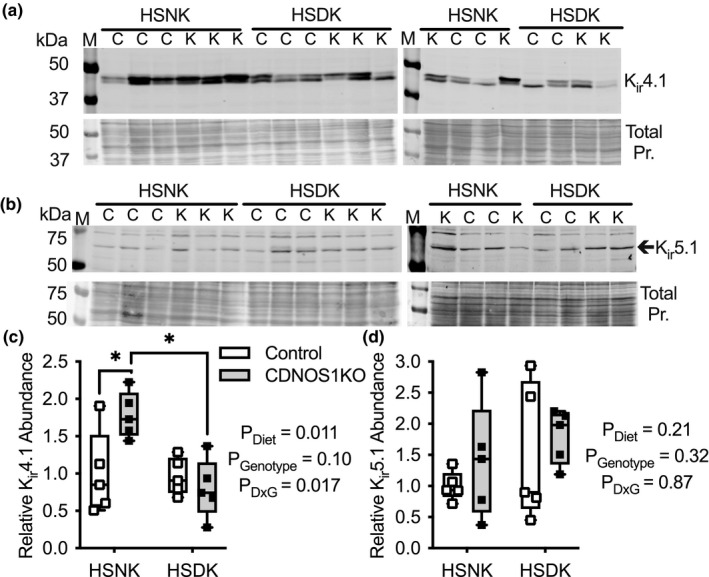
Renal cortical abundance of K_ir_4.1 and K_ir_5.1 channels from male control (C) and CDNOS1KO (K) mice on day 4 of a high Na^+^, normal K^+^ (HSNK; 1.6% Na^+^, 1% K^+^) diet or a high Na^+^, deficient K^+^ diet (HSDK; 1.6% Na, 0.0001% K^+^). (a) Western blots of K_ir_4.1 abundance, *N* = 5 animals per group. Total Protein (pr.) stained with Coomassie to demonstrate equal loading of samples. (b) Western blots of K_ir_5.1 abundance, *N* = 5 animals per group. (c) Relative to control HSNK, K_ir_4.1 and (d) K_ir_5.1 densitometry. Two‐Factor ANOVA reported, and * represents *p* < 0.05 from the Tukey's multiple comparison test. M‐ molecular marker, kDa‐ kilodalton

Finally, we measured K_ir_4.1/K_ir_5.1 cortical abundance from mice on the HSNK and HSDK diets (Figure 7). Similar to the results with a HSNK gel diet in Figure 4, CDNOS1KO mice had statistically greater cortical K_ir_4.1 abundance compared to controls, but this genotypic difference was not observed when the diet was deficient in K^+^ (Figure 7a,c). There were no statistical differences in K_ir_5.1 abundance between the mice or with a HSDK diet (Figure 7b,d). Thus, K_ir_4.1 upregulation may allow CDNOS1KO mice to maintain homeostasis of plasma potassium when consuming a potassium‐replete diet.

## 4 DISCUSSION

The CCD principal cell plays a critical role in the regulation of Na^+^ and K^+^ transport, thereby allowing the body to remain in homeostasis when intake is altered. High dietary NaCl is a stimulator of kidney NO in rodents (Hyndman et al., 2013; Mattson & Bellehumeur, 1996) and humans (Imanishi et al., 2013). We previously reported that NOS1β, the predominant enzyme that generates NO in the principal cell, is necessary to promote natriuresis and maintain extracellular fluid homeostasis when eating a high‐NaCl diet (Hyndman et al., 2013, 2015, 2017). Previously, Lu & Wang, 1996, demonstrated that ex vivo blockade of NOS in the CCD inhibited basolateral K^+^ conductance, which was restored by exogenous NO. Consistent with these results, we observed that in cultured cells transfected with K_ir_4.1/K_ir_5.1 and NOS1β inhibition of NOS1β activity with L‐NAME led to a significant decrease in the number of active channels. Surprisingly, however, K_ir_4.1 abundance and K_ir_4.1/K_ir_5.1 channel activity was increased in CDNOS1KO mouse CCD. This is likely due to the increased plasma aldosterone, as both spironolactone and a low‐K^+^ diet, which lowered aldosterone levels, normalized K_ir_4.1 abundance. Spironolactone treatment resulted in higher plasma K^+^ in high‐salt‐fed CDNOS1KO mice compared to controls indicating that increased plasma potassium in this mouse model drives aldosterone secretion to maintain potassium homeostasis. The elevated aldosterone in turn results in increased ENaC activity and is associated with higher plasma Na^+^ (Hyndman et al., 2015, 2017), which is reversed by spironolactone and low‐K^+^ diet. It should be noted, that the hypernatremia observed in the high salt‐fed CDNOS1KO mouse may also be a consequence of changes to arginine vasopressin and/or resetting of the osmostat as has been reported with mild hypernatremia (Gregoire, 1994).

The mechanisms by which the increased plasma potassium occurs in CDNOS1KO mouse requires further study but it is unlikely to be a primary defect in ENaC or K_ir_4.1/K_ir_5.1 activity, as these are both upregulated in the mutant mice compared to controls, suggesting compensation for a defect in other pathways. Interestingly, the ability to maintain potassium homeostasis appears to be specifically impaired during high‐Na^+^ diet, as CDNOS1KO mice fed normal Na^+^ do not have elevated aldosterone or hypernatremia. This suggests an abnormality in the mechanisms that uncouple Na^+^ and K^+^ homeostasis.

Previous studies with perfused CCD tubules from rabbits determined that chronic 6–10 day 11‐deoxycorticosterone (DOCA) treatment results in activation of basolateral Na^+^‐K^+^‐ATPase (Koeppen et al., 1983) and enhanced basolateral K^+^ conductance that enhances K^+^ secretion (Sansom et al., 1989). Consistent with the idea that K_ir_4.1/ K_ir_5.1 upregulation in response to increased aldosterone favors K^+^ secretion, Tomilin et al., 2018 demonstrated increased CCD principal cell K_ir_4.1/K_ir_5.1 activity in response to high‐potassium diet and treatment. Increased basolateral K^+^ channel activity should hyperpolarize the principal cell and increase Na^+^ reabsorption through ENaC to generate a more lumen‐negative transepithelial potential and drive K^+^ secretion. Consistent with this idea, pharmacological inhibition of K_ir_4.1/K_ir_5.1 activity in isolated CCD decreased ENaC activity (Isaeva et al., 2020). In addition, the study by Sansom et al. (1989) showed that in DOCA‐treated rabbits, basolateral K^+^ conductance is increased, and the driving force for K^+^ across the basolateral membrane favors K^+^ movement from plasma into the principal cell, which could further increase principal cell K^+^ secretion. On the other hand, mice in which *Kcnj10* was knocked out in the principal cell develop hypokalemia when fed a low‐K^+^ diet or with thiazide treatment because of an inability to downregulate ENaC and ROMK activity in the knockout mouse (Penton et al., 2020). A possible explanation for this is that depolarization in the principal cells of these mice stimulates intracellular signaling cascades that stimulate ENaC‐dependent K^+^ secretion (Sorensen et al., 2019). This is similar to the concept that K_ir_4.1/K_ir_5.1 serves as a potassium sensor in the DCT to regulate Na^+^ reabsorption through the sodium chloride cotransporter (Manis et al., 2020; Su et al., 2019) explaining hypokalemia observed in rodents and humans lacking functional K_ir_4.1/K_ir_5.1 channels (Celmina et al., 2019; Cuevas et al., 2017; Malik et al., 2018; Palygin, Levchenko, et al., 2017; Schlingmann et al., 2021; Su et al., 2019; Tomilin et al., 2018). Thus, the precise effects of changes in K_ir_4.1/K_ir_5.1 activity on K^+^ homeostasis likely depend on the degree of change as well as the affected nephron segment.

The renin‐angiotensin‐aldosterone system is typically downregulated when there is an increase in dietary NaCl but normal K^+^ intake. In the collecting duct, this results in decreased ENaC activity to limit Na^+^ absorption as well as K^+^ wasting. CCD principal cell K_ir_4.1/K_ir_5.1 activity is also reduced when mice are fed high‐Na^+^ diet (Tomilin et al., 2018). This is consistent with the idea that decreased circulating aldosterone during high Na^+^ intake should suppress K_ir_4.1/K_ir_5.1 activity, the opposite of the phenotype observed in the CDNOS1KO mice.

High‐salt diet also upregulates natriuretic pathways (e.g., NO, endothelin‐1, atrial natriuretic peptide). Indeed, an increase in Na^+^ delivery to the principal cell results in ENaC‐mediated sodium reabsorption and stimulation of NOS1β that acts in a negative feedback loop to inhibit ENaC and promote natriuresis (Hyndman et al., 2015). Although NO also stimulates K_ir_4.1/K_ir_5.1, as shown here and previously, (Lu et al., 1997; Lu & Wang, 1996) suppression of aldosterone‐dependent activation appears to dominate over NO stimulation under these conditions. This may be because the role of NO is in moment‐to‐moment coupling of apical ENaC activity and basolateral K_ir_4.1/K_ir_5.1 activity, as suggested by previous studies (Lu et al., 1997) rather than longer‐term control of K_ir_4.1/K_ir_5.1 activity. This also explains why we observe increased K_ir_4.1/K_ir_5.1 activity despite loss of principal cell NO in the CDNOS1KO mice, as circulating aldosterone is increased despite high‐Na^+^ diet. We previously reported that CDNOS1KO mice have inappropriately high circulating aldosterone on a chronic, grain‐based, and high‐NaCl diet (Hyndman et al., 2017). We confirmed this observation in the current study with mice on a chronic, purified, and high‐NaCl diet.

ROMK abundance was not significantly different in the principal cells but was statistically greater in the DCT CDNOS1KO mice compared to control mice, consistent with prior studies showing upregulation of ROMK in DCT2 in mice fed a high‐K^+^ diet (Wade et al., 2011). Interestingly, apical translocation of ROMK in DCT2 was also increased in aldosterone synthase knockout mice, suggesting ROMK regulation in DCT2 is at least in part aldosterone‐independent (Todkar et al., 2015). A limitation to our study is that we did not measure ROMK activity directly, but we speculate that this increased ROMK expression could facilitate K secretion in the CDNOS1KO mouse on a high‐NaCl diet.

There are some additional limitations to our study. Our design only tested normal and low dietary K^+^ thus, conclusions cannot be extrapolated to high dietary K^+^ handling in the CDNOS1KO mouse. Moreover, the K_ir_4.1/ K_ir_5.1 activity measurements were only from HSD‐fed mice and we did not measure K_ir_4.1 homomeric channel activity. One might predict that an increase in abundance of K_ir_4.1 would significantly affect both homomeric and heteromeric activity but this requires future experimentation.

**FIGURE 8:**
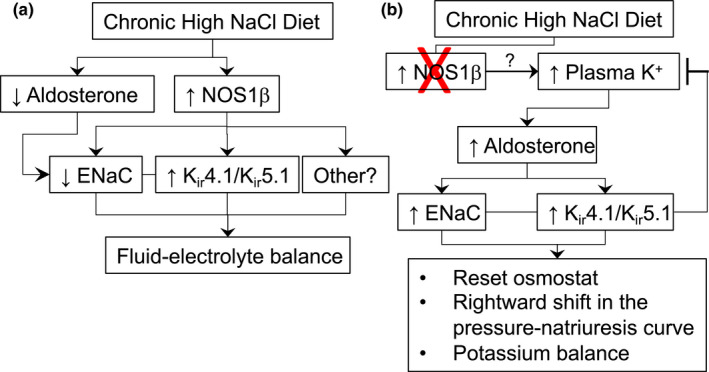
Hypothetical scheme of the proposed involvement of principal cell NOS1β in regulating Na^+^ and K^+^ balance in vivo during a chronic increase in dietary NaCl. (a) Physiologically, chronic high‐salt diet suppresses aldosterone thus reducing ENaC expression and activity and promoting natriuresis. Principal cell NOS1β is also activated leading to decreased ENaC activity and putative activation of basolateral K_ir_4.1/K_ir_5.1 to maintain fluid‐electrolyte balance. NOS1β may also stimulate other unknown pathways to maintain potassium secretion under conditions of suppressed aldosterone. (b) However, if principal cell NOS1β is not activated during high salt feeding there is a failure to appropriately suppress aldosterone, likely due in part to increased plasma K^+^ (that is, unmasked with spironolactone treatment), that then activates both ENaC and K_ir_4.1/K_ir_5.1, and overrides the decrease in K_ir_4.1/K_ir_5.1 activity expected from loss of NOS1β. Consequently, there is likely a resetting of the osmostat (high plasma Na^+^), a rightward‐shift in the pressure‐natriuresis curve (Hyndman et al., 2013), but plasma K^+^ is kept within the normal range. The mechanism by which plasma K^+^ is increased in NOS1β knockout animals remains to be determined

In conclusion, the loss of principal cell NOS1β likely impairs renal K^+^ secretion in the face of high‐Na^+^ diet, stimulating a compensatory increase in aldosterone, and upregulation of ENaC and the basolateral K_ir_4.1/K_ir_5.1 channel in the CCD principal cell (Figure 8). While this preserves K^+^ homeostasis, it comes at the expense of Na^+^ retention and likely resetting of the osmostat and hypertension (Hyndman et al., 2013, 2017). Although a high‐K^+^ diet typically has a blood pressure lowering effect and is recommended for patients with hypertension (Whelton et al., 2018), salt‐sensitive hypertension is associated with kidney NO deficiency both in rodent models like the CDNOS1KO (Hyndman et al., 2013) or the Dahl salt‐sensitive rat (Zou & Cowley, 1999) and in hypertensive, diabetic patients (Imanishi et al., 2013). Thus, in kidney NO deficiency, diets high in both Na^+^ and K^+^ may lead to excessive aldosterone production driving Na^+^ retention and/or unintentional hyperkalemia that further complicates and increases the risk of cardiovascular and kidney diseases.

## DISCLOSURES

None.

## AUTHOR CONTRIBUTIONS

K.A.H. and J.S.P. designed the study, K.A.H., E.I, O.P., and L.D.M. carried out experiments, K.A.H., E.I., A.R.R., O.P, A.S., and J.S.P. analyzed the data, made the figures, and drafted the manuscript. All authors approved the final version of the manuscript.
